# Development of a Screening Tool for Common Mental Disorders Among General Hospital Inpatients in China

**DOI:** 10.3389/fpsyt.2021.770255

**Published:** 2021-12-23

**Authors:** Shuai Yuan, Yizhong Shen, Jingwen Liu, Zilin Chen, Lijiao Zheng, Lihao Chen, Hanwei Chen, Huiqiang Feng, Hongbo He

**Affiliations:** ^1^Guangzhou Huiai Hospital, Affiliated Brain Hospital of Guangzhou Medical University, Guangzhou, China; ^2^Panyu Central Hospital, Guangzhou, China; ^3^Health Commission of Guangdong Province, Guangzhou, China

**Keywords:** general hospital, mental health, reliability, validity, PHQ-9, GAD-7, AIS, C-SSRS

## Abstract

**Background:** Depression and anxiety disorders are common conditions among general hospital inpatients, but are believed to be under-recognized in China.

**Methods:** A short, practical questionnaire termed the happiness index scale (HIS) was developed for screening co-morbid mental disorders in non-psychiatric clinical settings. The HIS was completed by 1,005 non-psychiatric inpatients in a general hospital in China. The reliability and validity of the HIS were then assessed.

**Results:** The HIS comprised eight items which loaded onto four dimensions: (a) sleep quality; (b) suicidal tendency; (c) depression; and (d) anxiety. These dimensions explained 84.2% of the total variance. Confirmatory factor analysis showed reasonably good fit of the four-factor model (χ^2^/df = 1.27, *p* < 0.001, goodness-of-fit index = 0.95, comparative fit index = 0.99, root-mean-square error of approximation = 0.008). The correlation coefficients between each item and the corresponding factor were all > 0.5. Cronbach's α of the entire scale was 0.83, indicating good internal consistency. The area under the ROC curve was 0.95 compared with the original 31-item scale. Using the optimal cut-off score of HIS (mild happiness), the sensitivity and specificity were 0.933 and 0.882, respectively.

**Conclusions:** The new HIS scale is a practical screening tool composed of eight items covering the four most common and important dimensions of mental disorder. The HIS exhibited good reliability and specificity. The HIS is potentially suitable for large-scale screening in busy non-psychiatric clinical settings in China. Further verification using larger samples is warranted.

## Background

In our rapidly developing society with its accelerated pace of life, psychological stress is an increasing concern. Mental and psychological problems have become the biggest health challenges faced by humans in the twenty-first century. Depression and anxiety disorders, which are mental disorders with high incidence rates, significantly affect patients' subjective feelings, physical health, and cognitive function and are often accompanied by symptoms of physical discomfort, attention disorders, or working memory and executive function abnormalities (1). In China, the prevalence of various mental diseases has increased from 2.7% in the 1950's to 17% at present and the incidence of medical condition with severe mental disorder is as high as 1%, which translates to about 16 million people (2). Severe mental disorders are six diseases stipulated in China, including schizophrenia, schizoaffective disorder, paranoid psychosis, bipolar disorder, mental disorders due to epilepsy, mental retardation accompanied by mental disorders. In addition, when it comes to the personalizing treatments of patients with severe mental disorders, the importance of measurement-based assessment and care *via* the use of instruments both psychometrically sound and amenable to implementation in practice is also noteworthy (3). Data on the overall prevalence of depression/anxiety disorders in China are scarce. A study conducted in a foreign country (4) used the Zung Depression Scale to screen for symptoms of depressive and anxiety disorders in outpatients with chronic diseases in general hospitals, and found that up to 64.7% of patients may have depression or borderline depression.

Hospitals receive a large number of patients with various diseases every day. As a special group, mental and psychological problems in inpatients are very common. Thus, most patients have “comorbidity” of physical disease and mental disorder. Depression and anxiety disorders are common mental disorders with physical symptoms that may cover up the subjective experience of depressive and anxiety disorders (5). More than two-thirds of patients with depression and/or anxiety initially visit a health care facility because of physical symptoms alone (6). Patients often go to the general outpatient department of a general hospital with chief complaints of somatic symptoms such as dizziness, headache, palpitation, chest pain, fatigue, insomnia, and abdominal pain rather than seeing a psychiatric specialist (7). More than half of patients attending primary care clinics have symptoms of anxiety, depression, or somatization. According to several surveys in China, about 25–40% of outpatients in general hospitals meet the diagnostic criteria for a mental disorder, which is 2–4 times higher than the rate in the general population (8, 9). According to further surveys in China, slightly more than 1/3 of outpatients in general hospitals suffer from somatic diseases, <1/3 suffer from neurosis, and the remaining 1/3 suffer from psychosomatic diseases (10–12). However, under the influence of the traditional biomedical model, most non-psychological specialists in general hospitals only pay attention to patients' bodies, ignoring their potential mental disorder. A prior study found that the recognition rate of psychological disorders in general hospitals is only 21% and that the treatment rate is only 10%. Not only does this waste medical resources, it also aggravates patients' economic and mental burdens while simultaneously causing tension between doctors and patients (13).

For many patients, the interval between the first episode and assessment at a psychiatric clinic is long (14), and it usually takes many years for a patient to get a correct diagnosis and treatment. Not only does this cause economic losses to the patient, it also increases the economic and spiritual burden of patients (15). A survey on misdiagnosis of mental disorder in general hospitals (16) found that the top five misdiagnoses are panic disorder at 100%, somatoform disorder at 81.6%, generalized anxiety disorder at 66.3%, comorbid anxiety and depression at 65.5%, and depression only at 38%. Furthermore, 6.3% of patients had been hospitalized due to misdiagnosis, they were hospitalized at the general hospital for mental disorder, of which 40% were hospitalized more than twice; one individual was hospitalized 10 times. Panic disorder results in 46.3% of hospital admissions due to misdiagnosis. The per capita outpatient misdiagnosis cost was estimated to be 3,900 yuan (611 dollars), and the per capita inpatient cost due to misdiagnosis was 9,980 yuan (1,563 dollars). The total misdiagnosis cost of the study subjects was 2,408,000 yuan (377,193 dollars), and the loss of resources is more than 2.4 billion yuan (4 billion dollars) if we use 1,000 hospitals in China for the calculation.

At present, many hospitals in China do not have a psychiatric department. Furthermore, about 50% of county hospitals do not have a psychiatrist. Most patients with mental disorder in remote areas must go to large cities for medical treatment. Mental health professionals in China have long been in short supply. The number of psychiatrists per 100,000 people in China is only one-fifth of that in developed countries and lower than the global average (17).

For general hospital patients with increased rates of depressive and anxiety disorders symptoms, general hospital physicians tend to be their earliest contacts. However, general hospital doctors lack working experience in the diagnosis and treatment of mental disorder—especially the ability to recognize somatic symptoms (18). Therefore, such patients are often missed or misdiagnosed, which leads to delayed treatment. Such delays can lead to a series of problems, the most serious of which is an increase in suicide risk.

The recognition and treatment of mental disorder is receiving increasing attention from the state, such as Document No. 77 of the 2016 National Health and Disease Control Development: Guidance on Strengthening Mental Health Services. In 2019, the Health Commission of Guangdong Province issued Document No. 78: Notice of Implementation Opinions on Strengthening the Construction of Social Psychological Service System in Guangdong Province. In 2021, Guangdong Province officially began to implement the “Guangdong Happiness Hospital (GHH) Project.” The aim of the project is to pay attention not only to patients' diseases, but also to their psychological well-being in clinical practice, strengthen psychological interventions for patients, and improve patients' medical experiences so as to achieve better medical results. The first step of the GHH Project is the development of a preliminary screening tool to quickly identify patients in need of intervention. The second step is to select a group of health care workers who are trained in psychological communication skills who can treat patients' physical problems and address their psychological needs. The third step is to conduct psychological and drug interventions with the patients identified using the new screening tool.

The purpose of the present study was to achieve the first step of the GHH Project by creating a preliminary screening tool. To do so, we extracted items from existing scales, namely The Patient Health Questionnaire Self-Rating Depression Scale (PHQ-9)/ Generalized Anxiety Disorder Screener (GAD-7)/ Athens Insomnia Scale (AIS)/ Columbia Suicide Severity Rating Scale (C-SSRS), to form a simplified scale called the Happiness Index Scale (HIS). The development of HIS can quickly screen the mental problems of inpatients in general hospitals, improve the rate of diagnosis of mental diseases, facilitate the rational allocation of medical resources, help patients get more standardized and efficient treatment, and help save medical costs. The development of HIS is the basis for the advancement of GHH, it provides patients, their families and medical staff with comprehensive psychological services, including psychological counseling, psychological assistance and other mental health services.

## Methods

### Sample

A cross-sectional study of 916 non-psychiatric inpatients was conducted from February 4 to February 5, 2021 in a third class general hospital in Guangzhou. Third Class General Hospital is a medical institution classified in accordance with the current Administrative Measures for Hospital Classification in China, which is the highest level in China. With more than 501 beds, it is a regional or higher hospital that provides high-level specialized medical and health services and performs higher education and scientific research tasks in several regions. The inclusion criteria were: clear awareness; the ability to understand the content of the questionnaire; and the ability to perform self-evaluation. The exclusion criteria were: a consciousness disorder; severe hearing or vision impairment; inability to understand the content of the questionnaire; a lack of self-evaluation ability; and patients in the intensive care unit (ICU) or operating room.

### Assessment Tools

Consisting of the Chinese version of the PHQ-9, GAD-7, AIS, and C-SSRS were used as an evaluation tool (thirty-one items total) to develop general demographic data questionnaires addressing gender, age, education level, marital status, and hospitalization details. The “questionnaire star” platform was used to assess patients within 24 h of admission. The starting part of the questionnaire is the informed consent section. Patients who provided informed consent can continue to answer the questions, while patients who refuse informed consent can skip the questionnaire items and submit scale directly. We received 916 valid questionnaires eventually. The research protocol was approved by the Ethics Committee of the Brain Hospital Affiliated to Guangzhou Medical University.

Patient Health Questionnaire Self-Rating Depression Scale (PHQ-9): A 9-item self-assessment tool for major depressive disorder (MDD) based on the Diagnostic and Statistical Manual of Mental Disorders, Fourth Edition (DSM-IV). It has good reliability and validity for depression assessment in inpatients in general hospitals in China (19).

Generalized Anxiety Disorder Screener (GAD-7): The first 13 items were selected, including 4 items from the existing anxiety scale plus nine items about GAD based on the DSM-IV. The 7 items with the greatest correlation were selected from the 13 items. The area under seven working curves (0.906) was very similar to the area under 13 working curves, and shows good reliability and validity when applied for anxiety assessment in inpatients in general hospitals in China (20).

Athens Insomnia Scale (AIS): This scale was designed in 1985 by Ohio State University School of Medicine and consists of eight items. The first five items address sleep induction, waking up at night, getting up late, total sleep time, and sleep quality. AIS has high consistency, reliability, and validity (21).

Columbia Suicide Severity Rating Scale (C-SSRS): Developed at Columbia University by Philips et al. (22), this scale has good specificity and sensitivity for suicide screening. The scale consists of seven items with responses of “yes” or “no” to determine whether a patient has suicidal ideation and suicidal behavior. According to the C-SSRS, those with a suicide plan who were ready to implement it within the past 1 month and those who had a suicide plan in the past 3 months have a high risk; those with a suicide plan but did not plan to implement it in the past 1 month have a moderate risk; and those who have suicidal ideas but no suicide plan in the past 1 month have a low risk.

### Classification of the Severity

Psychological problem severity classification: mild: PHQ - 9 scored were 5–9 points; Gad-7 scored were 5–9 points; AIS scores were 6–9 points; C-SSRS have no risk of suicidal ideation or behavior. Moderate: PHQ-9 scored were 10–14 points; GAD - 7 scored were 10-13 points; AIS scores of 10–15; C-SSRS have low risk of suicidal ideation and no risk of suicidal behavior. Severe: PHQ - 9 scored were 15 points or more; Gad-7 scored were 14 points or more; AIS scored were 16 points or more; C - SSRS have moderate risk of suicidal ideation or above, have moderate risk of suicidal behavior or above. If two risk scales are met at the same time, the higher risk shall prevail (for example, if both moderate and severe conditions are met, it is severe).

### Statistical Analysis

The survey data were exported from Questionnaire Star to Excel. A SPSS database was then established. We used SPSS version 22.0 and Amos 22.0 for statistical analysis. All count data and the constituent ratio and utilization rate were statistically described. We randomly divided 916 patients into two subgroups. Half of patients were used for exploratory factor analysis, and the correlation of the 31 items in the four scales was evaluated by Spearman correlation analysis. The Kaiser-Meyer-Olkin measure and Bartlett's sphericity test were used to verify whether the data are suitable for exploratory factor analysis. The principal component analysis (PCA) method and maximal variation direct axis methods were used in this study to determine the number of factors. Varimax orthogonal rotation was then used to determine the factor structure. The results of the factor analysis were used to identify the most robust items which were retained for the final version of the scale. The items in the final version of the scale were then assessed by a confirmatory factor analysis using the second half of the sample. The fit of the model was evaluated using the following indices: the ratio of chi-squared to the degrees of freedom (χ^2^/df), goodness-of-fit index (GFI), comparative fit index (CFI), and root-mean-square error of approximation (RMSEA). The internal consistency of the final version of the HIS was evaluated by Cronbach's α coefficient and the content validity was evaluated by assessing the correlation of the different factors of the scale using Spearman correlation analysis. Test-retest reliability, Kappa consistency test, and Guttman split-half coefficient were also used to evaluate the reliability of the HIS. A *p*-value < 0.05 was considered statistically significant. Determination of the gauge boundary value was performed by receiver operating characteristic (ROC) curve analysis.

## Results

### Patient Characteristics

Among the 916 cases, 460 (50.2%) were male. Regarding age, 540 (58.9%) were under 60 years old. The median age of the sample was 53 years old. Regarding marital status, 834 patients (91.0%) were married. See Table 1 for more details.

**Table 1:** Demographic characteristics of the respondents.

**Project**	**Total sample (***N*** = 916)**	**Percentage %**
**Gender**		
Male	460	50.2
Female	456	49.8
**Age**		
<60 years old	540	58.9
≥60 years old	376	41.1
**Degree of education**	
Junior high school and below	601	65.6
Senior high school to junior college	236	25.8
Bachelor's degree or above	79	8.6
**Marital status**		
Married	834	91.0
Unmarried	82	9.0
**Inpatient department**		
Internal medicine	416	45.4
Surgery	312	34.1
Obstetrics and Gynecology Department	99	10.8
Otolaryngology	45	4.9
Unclear^a^	44	4.8

a *The inpatient department was not clearly stated*.

### Exploratory Factor Analysis

We performed Spearman correlation analysis for the 31 items of the four scales. We found that there were seven items from the C-SSRS. The correlation coefficient between item 3 and item 4 is 0.942, and the correlation coefficient between item 6 and item 7 is 1.000. Therefore, we deleted one of the two pairs of items (Article 4 and Article 7) whose correlation coefficient is >0.9. This left 29 items in the four scales. Exploratory factor analysis (23, 24) was conducted using data from a random sample of 458 patients (half of the total patients). Both the Kaiser-Meyer-Olkin measure (KMO = 0.791) and Bartlett's sphericity test (3,043.547, *p* < 0.001) suggested that the data are suitable for exploratory factor analysis. We used the results of the exploratory factor analysis to remove items that met any of the following criteria (25): (a) items with a factor loading >0.80 on all factors; and (b) items loading on more than one factor with a factor loading ≥ 0.40. Based on these criteria, 23 items were deleted. The remaining 8 items that made up the final Happiness Index Scale (HIS) are listed in Table 2.

**Table 2:** Results of exploratory factor analysis (among 458 patients) of the 4-factor model of the Happiness Index Scale (HIS).

**Items**	**Factor loadings**			
	**1**	**2**	**3**	**4**
**Factor1: Sleep quality**				
ais-4.Total sleep time	**0.913**	0.027	0.167	0.203
ais-5.Total sleep quality (no matter how long you sleep)	**0.902**	0.023	0.213	0.199
**Factor2: Suicidal tendency**				
cre-3.Have you been thinking about how to kill yourself?	0.024	**0.930**	0.036	−0.016
Cre-2.Do you actually have some ideas about suicide?	0.022	**0.921**	0.065	0.103
**Factor3: Depression trend**				
phq-1. Little interest or pleasure in doing things	0.179	0.024	**0.879**	0.162
phq-2.Feeling down, depressed, or hopeless	0.195	0.093	**0.811**	0.286
**Factor4: Degree of anxiety**				
gad-6.Becoming easily annoyed or irritable	0.206	0.037	0.175	**0.854**
gad-3. Worrying too much about different things	0.186	0.054	0.258	**0.822**
**Eigenvalue of the factor**	**3.30**	**1.68**	**0.98**	**0.79**
**Percent of total variance accounted for by the factor**	**22.4%**	**21.6%**	**20.1%**	**20.1%**

### Factor Structure and Internal Consistency of the HIS

As shown in Table 2, Factor 1 includes two items from the AIS related to sleep quality; Factor 2 includes two items from the C-SSRS related to suicidal thoughts; Factor 3 includes two items from the PHQ related to the core items of depression; and Factor 4 includes two items from the GAD-7 related to anxiety.

The results of the confirmatory factor analysis (26) of the eight items using the responses from the second half of the sample (458 patients) based on varimax rotation of the factors is shown in Table 2. The eigenvalues of the five factors varied from 0.79 to 3.30 and the four factors explained 84.2% of the total variance in the results. The items all loaded on the expected factors with a minimum loading of 0.81. The model fit parameters were satisfactory: χ^2^/df = 1.27, *p* < 0.001; GFI = 0.95; CFI = 0.99; and RMSEA = 0.008.

The correlation coefficient ranged from 0.371 to 0.636 for each dimension score of the scale, and from 0.046 to 0.511 between the dimension and total score, as shown in Table 3. In the exploratory factor analysis, the KMO value was 0.791. For Bartlett's sphericity test *p* < 0.001, which was a significant level, suggesting the data could be used for factor analysis. In addition, the principal component analysis (PCA) method and maximal variation direct axis methods were used in this study. The number of fixed factors was 4 and the extracted four common factors had eigenvalues > 1 which explained 84.2% of the total variation.

**Table 3:** Correlations between each of the four dimension of the Happiness Index Scale (HIS) and between the dimension (factor) and the total score of the original scale.

**Dimension**	**Factor1**		**Factor2**		**Factor3**		**Factor4**	
	***r***	***p***	***r***	***p***	***r***	***p***	***r***	***p***
Factor1	1.000	-						
Factor2	0.045	0.337	1.000	-				
Factor3	0.482	**<0.001**	0.046	0.327	1.000	-		
Factor4	0.511	**<0.001**	0.070	0.133	0,489	**<0.001**	1.000	-
Total score	0.893	**<0.001**	0.713	**<0.001**	0.783	**<0.001**	0.896	<0.001

*Factor1: Sleep quality; Factor2: Suicidal tendency; Factor3: Depression trend; Factor4: Degree of anxiety. Correlation coefficient is Spearman's r*.

### Reliability

There were 32 participants who were administered the HIS twice (2–3 weeks apart) to evaluate the test-retest reliability. The correlation coefficient of the two scores was 0.70 (*p* < 0.001), which indicated good reliability.

For the Kappa consistency test, the Kappa value was 0.545. The original scale screened 154 people with severe mental problems, while the simplified HIS scale screened 288 people with severe mental problems. Among them, 138 people with severe mental problems were screened in the same way with the two scales. The simplified scale achieved the “gold standard” (the PHQ-9/GAD-7/AIS/C-SSRS) and the consistency of the screening rate of severe mental problems was = 138/154 = 89.6%. The overall screening consistency rate = 2,776+314+92+138) /4,193 = 79.2%. See Table 4 for additional details.

**Table 4:** Kappa consistency test for the Happiness Index Scale (HIS).

		**Severity of original scale**						
		**Negative**	**Mild**	**Moderate**	**Severe**	**Total**	**Kappa value**	***p*** **-value**
**Severity of HIS and original scale cross-tabulated**								
Severity of the Happiness Index Scale (HIS)	Negative	2,776	42	23	7	2,848	0.545	0.000
	Mild	352	314	22	4	692		
	Moderate	21	247	92	5	365		
	severe	0	26	124	138	288		
**Total**		3,149	629	261	154	4,193		

Cronbach's α was 0.83 for all 8 items and 0.80, 0.81, 0.89, and 0.71, respectively, for the four dimensions of the scale. All values were >0.7, indicating good internal consistency of the scale and its various dimensions (27). The Guttman split-half coefficient was 0.68, which also proved that the scale has high reliability.

### Best Critical Value, Sensitivity, and Misjudgment Rate of the Severity of Psychological Problems

**Figure 1:**
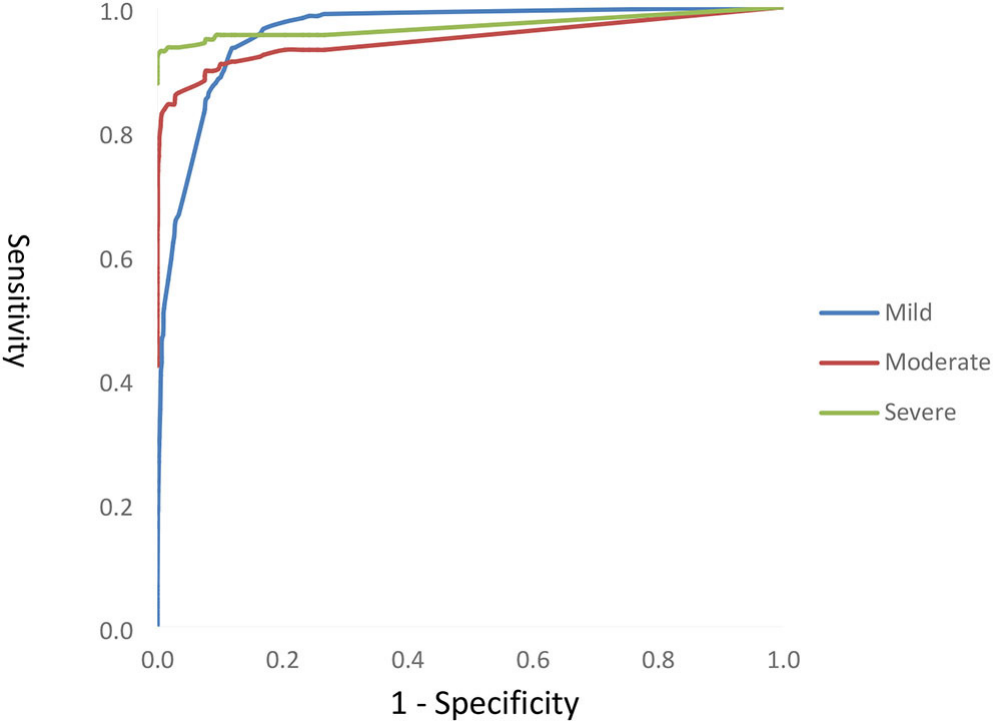
ROC curve analysis.

The ROC curve was drawn to evaluate the diagnostic value of the screening tool. The area under the curve (AUC) values of mild, moderate, and severe psychological problems were 0.960, 0.921, and 0.955, respectively (all *p* < 0.01), which indicate that the diagnosis ability is better. Because the scoring method for each item is not the same (e.g., the suicide dimension is answered as “yes/no,” while the other dimensions are not), we need to calculate the weight of each item so that we can calculate the total score and cutoff value of the scale. According to the weight of each item, the total score of the scale was 6.247. The optimal operating point (OPP) (28) was determined by the maximum sensitivity, specificity, and Youden index. When the cutoff value of mild psychological problems was 0.365, the sensitivity, specificity, and Youden index were the highest and the screening ability was the best; when the cutoff value of moderate psychological problems was 0.955, the sensitivity, specificity, and Youden index were the highest and the screening ability was the best; and when the cutoff value of severe psychological problems was 1.470, the sensitivity, specificity, and Youden index were the highest and the screening ability was the best. See Figure 1 for additional details. Lastly, the classification criteria of the new version of the scale were determined to be: < 0.365, no psychological problems; 0.365–0.955 or =0.365, mild psychological problems; 0.955–1.470 or =0.955, moderate psychological problems; and > or =1.470, severe psychological problems.

## Discussion

The present study aimed to develop a new screening tool for depressive and anxiety disorders by the selection of items from a series of clinical tests and modern statistical techniques. The newly developed screening tool, which we termed HIS, can assess the core features of depressive and anxiety disorders based on items optimized using factor analysis. It is important to note that HIS is only a screening tool, and that therefore a full clinical examination by a full licensed psychiatrist is needed in order to provide a specific diagnosis and eventually to treat the patients. To evaluate the reliability and validity of HIS, the current study used data from 916 non-psychiatric inpatients in Guangzhou, China (who completed the full 31-item questionnaire) to develop a much briefer 8-item version of the scale (HIS). Extensive evaluation showed that this scale has good internal validity and that its four dimensions account for 84.2% of the total variance and are relatively independent of each other. The correlation coefficients between each dimension score and the total score were greater than the dimensions themselves, indicating good content validity (29). Furthermore, confirmatory factor analysis indicated that the data fit the four-factor model reasonably well.

According to our results, the HIS has four dimensions: (a) sleep quality, (b) suicidal tendency, (c) depression trend, and (d) degree of anxiety. Among them, the depressive and anxiety disorders dimensions both contain the core terms for diagnosing Depression and Generalized Anxiety Disorder (according to ICD-10 criteria (30)); the sleep dimension contains questions about sleep duration and total sleep quality, which seem to be highly generalized terms; and the suicide dimension has two items on suicidal thoughts. The HIS thus appears reasonable based on its content.

The HIS was also found to have good reliability, with a Cronbach's α coefficient of 0.83 and a Guttman split-half coefficient of 0.68. When Cronbach's α coefficient and the split-half reliability are > 0.6, a scale is generally believed to have good reliability (31–33). Furthermore, the Kappa value was 0.545 and the test-retest reliability was 0.70, indicating that the internal consistency and measurement stability of HIS are good.

For structural validity, when the correlation coefficient between the dimensions and the total score is ~0.3–0.8, we generally believe that the scale has good structure validity (34). The correlation coefficients of each dimension and the total score ranged from 0.713 to 0.896. It is believed that if the common factors obtained from the scale explain more than 50% of the variance then the scale has good structural validity (35). In the present study, four common factors with eigenvalues > 1 were extracted by the maximal variation direct intersection axis method, which explained 84.2% of the total variation. Moreover, the items were all > 0.4 on the corresponding factor loading value, indicating good structural validity.

Depressive and anxiety disorders are generally considered distinct entities in psychiatric practice (36). However, as they are closely linked or highly comorbid (37, 38), it is difficult to distinguish between depressive and anxiety disorders using self-reported scales (39, 40). Therefore, we developed HIS to obtain a single score about general distress that summarizes the effects of depressive and anxiety disorders, rather than one that differentiates these mood construct, that is short and practical for screening purposes. In practice, a more detailed assessment is required for patients who score high on the HIS. That is why we note that HIS is most appropriate as a preliminary screening tool. Obviously, there are many scales to evaluate patients' depression, anxiety and other aspects, but they only evaluate one dimension. In general hospitals, it would take a lot of time for patients to complete all the scales. It would be nice if we could develop a scale with different dimensions that didn't take too long to complete. This is also the original intention of our development of HIS. HIS is simple and efficient, and can effectively detect patients requiring further intervention. It takes <30 s to finish HIS. Of course, as mentioned above, it is only a preliminary screening tool. But HIS is undoubtedly of great help to the detection rate of psychological problems in general hospitals.

## Limitations

This study is subject to several limitations. Firstly, our data were collected at a general hospital. Different from psychiatric departments, the number of inpatients in non-psychiatric departments with suicidal ideas and behaviors was small, which may lead to inaccurate items being retained in the suicide dimension. Secondly, due to the limited sample size, separate analyses of inpatients from different departments was not performed. Since all of the participants were recruited from one general hospital in Guangzhou, China, caution should be taken when generalizing the findings to other clinical settings or the whole country. Thirdly, only diagnoses based on the original 31-item scale were used as the “gold standard” to assess the criterion validity of HIS. Other aspects of validity, such as the concurrent validity (i.e., correlations with other established measures), should be tested in future research. Fourthly, when selecting items, we did not conduct the exploratory factor analysis and confirmatory factor analysis again with the other half of the sample, although the use of expert group review and test of several items with similar meaning to select the optimized items is expected to decrease this limitation.

The brief 8-item scale developed in this study showed good psychometric properties. Thus, further development of this scale in China is warranted and simpler scales and scoring patterns can be explored.

In summary, in the present study we developed and validated the HIS to screen depressive and anxiety disorders in non-psychiatric clinical settings in China. Although it showed satisfied reliability and validity, further studies are needed in different Chinese populations to evaluate the questionnaire, including determination of norms and hierarchical clusters to establish a hierarchical management model for patients screened by the HIS.

## Data Availability Statement

The original contributions presented in the study are included in the article/Supplementary Materials, further inquiries can be directed to the corresponding author/s.

## Author Contributions

All authors listed have made a substantial, direct, and intellectual contribution to the work and approved it for publication.

## Conflict of Interest

The authors declare that the research was conducted in the absence of any commercial or financial relationships that could be construed as a potential conflict of interest.

## Publisher's Note

All claims expressed in this article are solely those of the authors and do not necessarily represent those of their affiliated organizations, or those of the publisher, the editors and the reviewers. Any product that may be evaluated in this article, or claim that may be made by its manufacturer, is not guaranteed or endorsed by the publisher.
