# Supplementary material for: Development of a Screening Tool for Common Mental Disorders Among General Hospital Inpatients in China
Source: Front Psychiatry. 2021 Dec 23;12:770255. doi: 10.3389/fpsyt.2021.770255 (PMC8732762; doi:10.3389/fpsyt.2021.770255)
Supplement: Supplementary file 1 [file Data_Sheet_1.PDF]

PHQ-9 抑郁症筛查量表

| 序号 | 项目 | 没有 | 有几天 | 一半以上时间 | 几乎天天 |
|---|---|---|---|---|---|
| 1 | 做事时提不起劲或没有兴趣 | 0 | 1 | 2 | 3 |
| 2 | 感到心情低落，沮丧或绝望 | 0 | 1 | 2 | 3 |
| 3 | 入睡困难、睡不安稳或睡得过多 | 0 | 1 | 2 | 3 |
| 4 | 感觉疲倦或没有活力 | 0 | 1 | 2 | 3 |
| 5 | 食欲不振或吃太多 | 0 | 1 | 2 | 3 |
| 6 | 觉得自己很糟或觉得自己很失败，或让自己、家人失望 | 0 | 1 | 2 | 3 |
| 7 | 对事物专注有困难，例如看报纸或看电视时 | 0 | 1 | 2 | 3 |
| 8 | 行动或说话速度缓慢到别人已经察觉？或刚好相反——变得比平日更烦躁或坐立不安，动来动去 | 0 | 1 | 2 | 3 |
| 9 | 有不如死掉或用某种方式伤害自己的念头 | 0 | 1 | 2 | 3 |

计分规则　0-4 分：没有忧郁症(注意自我保重)

5-9 分：可能有轻微抑郁症(建议咨询心理医生或精神科医生)

10-14 分：可能有中度抑郁症(最好咨询心理医生或精神科医生)

15-19 分：可能有中重度抑郁症(建议咨询心理医生或精神科医生)

20-27 分：可能有重度抑郁症(一定要看心理医生或精神科医生)

核心项目　项目 1.4.9 任何一题得分＞1 分，即选择 2.3 需要关注

项目 1.4 代表抑郁核心症状，项目 9 代表自伤观念

GAD-7 焦虑症筛查量表

| 序号 | 项目 | 没有 | 有几天 | 一半以上时间 | 几乎天天 |
|---|---|---|---|---|---|
| 1 | 感到紧张、焦虑或急切 | 0 | 1 | 2 | 3 |
| 2 | 不能够停止或控制担忧 | 0 | 1 | 2 | 3 |
| 3 | 对各种各样的事情担忧过多 | 0 | 1 | 2 | 3 |
| 4 | 很难放松下来 | 0 | 1 | 2 | 3 |

| | | | | | |
|---|---|---|---|---|---|
| 5 | 由于不安而无法静坐 | 0 | 1 | 2 | 3 |
| 6 | 变得容易烦恼或急躁 | 0 | 1 | 2 | 3 |
| 7 | 感到似有害怕的事情发生而害怕 | 0 | 1 | 2 | 3 |

计分规则 0-4 没有焦虑症 （注意自我保重）
5-9 可能有轻微焦虑症 (建议咨询心理医生或心理医学工作者)
10-13 可能有中度焦虑症, (最好咨询心理医生或心理医学工作者)
14-18 可能有中重度焦虑症 (建议咨询心理医生或精神科医生)
19-21 可能有重度焦虑症 (一定要看心理医生或精神科医生)

阿森斯失眠量表（Athens Insomnia Scale,AIS）

本量表用于记录您对遇到过的睡眠障碍的自我评估。 对于以下列出的问题， 如果在过去 1 个月内每星期至少发生 3 次在您身上， 就请您圈点相应的自我评估结果。

| 序号 | 项目 | 0 | 1 | 2 | 3 |
|---|---|---|---|---|---|
| 1 | 入睡时间（关灯后到睡着的时间） | 没问题 | 轻微延迟 | 显著延迟 | 延迟严重或没有睡觉 |
| 2 | 夜间苏醒 | 没问题 | 轻微影响 | 显著影响 | 严重影响或没有睡觉 |
| 3 | 比期望的时间早醒 | 没问题 | 轻微提早 | 显著提早 | 严重提早或没有睡觉 |
| 4 | 总睡眠时间 | 没问题 | 轻微不足 | 显著不足 | 严重不足或没有睡觉 |
| 5 | 总睡眠质量（无论睡多长） | 满意 | 轻微不满 | 显著不满 | 严重不满或没有睡觉 |
| 6 | 白天情绪 | 正常 | 轻微低落 | 显著低落 | 严重低落 |
| 7 | 白天身体功能（体力或精神：如记忆力、 认知力或注意力等） | 足够 | 轻微影响 | 显著影响 | 严重影响 |
| 8 | 白天思睡 | 无思睡 | 轻微思睡 | 显著思睡 | 严重思睡 |

计分规则 0~5 分: 无睡眠障碍
6~9 分：轻度失眠
10~15 分：中度失眠
16~24 分：重度失眠

哥伦比亚-自杀严重程度评定量表（C-SSRS）

| 自杀意念 | |
|---|---|
| 问题 1 和 2， 如果对这两个问题的回答均为否， 请转至“自杀行为” 部分， 如果对问题 2 的回答为“是”， 请询问问题 3、 4、 5，如果对问题 1 和/或 2 的回答为“是”， 请完成下面的“意念强度”部分。 | 自上次访问以后 |
| 1.希望死去<br>你曾希望自己死去或者希望自己睡着后不再醒来吗？<br>如果是， 请描述： | 是 否<br>□ □ |
| 2. 不具体的主动自杀想法<br>您确实有过自杀的想法吗？ | 是 否 |

| | |
|---|---|
| 如果是， 请描述： | □ □ |
| 3. 有方法（非计划） 但无行动意图的主动自杀意念<br>您想过会如何自杀吗？<br>如果是， 请描述： | 是 否<br><br>□ □ |
| 4. 有行动意图但无具体计划的主动自杀意念<br>您有过这些想法并有过实施这些想法的意图吗？<br>如果是， 请描述： | 是 否<br><br>□ □ |
| 5. 有具体计划和意图的主动自杀意念<br>您已经开始制定或者已经制定了详细的自杀计划吗？ 您是否想执行这个计划？<br>如果是， 请描述： | 是 否<br><br>□ □ |
| 意念的强度 | |
| 以下各项按照最强烈的意念类型（即： 下面的 1-5， 其中 1 为最不强烈， 5 为最强烈） 来评定。<br>最强烈的意念： 类型编号（1-5） 意念描述 | 意念强烈程度 |
| 频率<br>您产生这些想法的次数有多少？<br>（1） 一周少于一次 （2） 一周一次 （3） 一周 2-5 次 （4） 每天一次或几乎每天一次 （5） 一天多次 | |
| 持续时间<br>当您有自杀想法时， 这些想法会持续多久？<br>（1） 短暂的时间-仅仅几秒或几分钟 （2） 不到 1 小时/一段时间<br>（3） 1-4 小时/很长时间 （4） 4-8 小时/几乎一天 （5） 超过 8 小时/持久或连续的时间 | |
| 可控性<br>如果您愿意的话， 您能否停止自杀或希望死去的想法？<br>（1） 能轻易地控制这些想法 （2） 能控制这些想法， 几乎没有困难 （3） 能控制这些想法， 但有些困难 （4） 能控制这些想法， 但非常困难 （5） 不能控制这些想法 （0） 不想控制这些想法 | |
| 制止因素<br>有没有什么因素-某人或某物（如： 家庭、 宗教、 死亡的痛苦等） -曾制止您产生希望死去的念头或实施自杀的想法？<br>（1） 制止因素的确曾制止过您的自杀企图 （2） 制止因素可能制止过您 （3） 不确定制止因素是否制止过您 （4） 制止因素很可能没用制止过您 （5） 制止因素绝对没有制止过您 （0） 不符合 | |
| 自杀意念的产生原因<br>哪些原因使您希望自己死去或自杀？ 是为了结束痛苦或终止您当时的感受（换句话说， 这种痛苦或您当时的感受使您无法继续忍受）， 还是为了引起他人的关注， 反应或报复他人？ 或者两者都有？<br>（1） 完全是为了引起他人的关注， 反应或报复他人 （2） 主要是为了引起他人的关注 （3） 既是为了引起他人的关注， 反应或报复他人， 也是为了结束/停止痛苦 （4） 主要是为了结束或停止痛苦（5） 完全是为了结束或停止痛苦（0） 不符合 | |
| 自杀行为 | |

| | |
|---|---|
| 实际尝试<br>您做过自杀尝试吗？<br>您做过伤害自己的事情吗？<br>您做过可能导致自己死亡的危险的事情吗？<br>您做了什么事情？<br>您曾将　　　作为结束您生命的方式吗？<br>当您　　　　时，您想过死（即使只有一点这样的想法）吗？<br>当您　　　　时，您曾试图结束自己的生命吗？<br>或者您想过您可能会因　　　而死吗？<br>或者您这样做纯粹是因为其他原因/没有任何自杀意图（如：为了释放压力、感觉好些、获得同情，或者使其他的事情发生）吗？（没有自杀意图的自伤行为）<br>如果是，请描述：<br>受试者有过非自杀性的自伤行为吗？ | 是 否<br>□ □<br>总的尝试次数<br>是 否<br>□ □ |
| 被中断的尝试：<br>是否有一次您开始做某事以便结束自己的生命时，但某人或某物在您真正开始前就阻止了您呢？<br>如果是，请描述： | 是 否<br>□ □<br>总的被中断次数 |
| 放弃的尝试：<br>是否有一次您开始做某事或试图结束自己的生命时，但在真正开始前您就停止了这样的行为呢？<br>如果是，请描述： | 是 否<br>□ □<br>总的放弃次数 |
| 预备的行动或行为：<br>您为自杀尝试或准备自杀采取了行动（比如：收集药品，获取枪支，将贵重物品送人或者写自杀遗言）吗？<br>如果是，请描述： | 是 否<br>□ □ |
| 自杀行为：<br>在评估期间发生了自杀行为？ | 是 否<br>□ □ |
| 完成的自杀： | 是 否<br>□ □ |
| 只对实际自杀尝试作答 | 最致命的自杀尝试的日期： |
| 实际的致命性/身体损伤：<br>0. 没有身体损伤或很轻的身体损伤（如：表皮擦伤）。 | |

| | |
|---|---|
| 1. 轻度身体损伤（如： 说话无精打采、 1 度烧伤、 轻度出血、 扭伤）。<br>2. 中度身体损伤： 需要就医（如： 有意识但困乏、 有些反应、 2 度烧伤、 大血管出血）。<br>3. 中重度身体损伤： 需要住院治疗并可能接受重症监护（如： 处于昏睡状态但反射完好、 不足身体面积 20%的 3 度烧伤、 大量失血但能恢复、 严重骨折）。<br>4. 重度身体损伤， 需要住院治疗并接受重症监护（如： 处于昏睡状态并失去反射功能、 超过身体面积 20%的 3 度烧伤、 大量失血并且生命体征不稳定、 要害部位的严重损伤）。<br>5.死亡 | |
| 潜在的致命性： 只在实际致命性-0 时才回答<br>实际自杀尝试在未造成身体损伤时的可能致命性<br>0. 行为可能不会造成伤害<br>1. 行为可能造成伤害但不会导致死亡<br>2. 尽管有医疗救护但行为仍可能导致死亡 | |

C-SSRS 评定近 1 个月内有自杀计划并准备实施者近 3 个月内有自杀行为者存在高度风险 近 1 个月内有自杀计划但未实施者存在中度风险近 1 个月内有自杀意念而无自杀计划者存在低度风险
